# Diagnostic Value of High-Resolution Ultrasound for the Evaluation of Capsular Width in Temporomandibular Joint Effusion

**DOI:** 10.3390/life12040477

**Published:** 2022-03-25

**Authors:** Daniel Talmaceanu, Lavinia Manuela Lenghel, Csaba Csutak, Nicolae Bolog, Daniel-Corneliu Leucuta, Horatiu Rotar, Ioan Tig, Smaranda Buduru

**Affiliations:** 1 Stomestet Dental Clinic, Department of Cranio-Maxillofacial Surgery, “Iuliu Haţieganu” University of Medicine and Pharmacy, 400372 Cluj-Napoca, Romania; daniel@stomestet.ro; 2 Department of Radiology, “Iuliu Haţieganu” University of Medicine and Pharmacy, 400006 Cluj-Napoca, Romania; pop.lavinia@umfcluj.ro (L.M.L.); csutakcsaba@elearn.umfcluj.ro (C.C.); 3 Phoenix Swiss Med GmbH, 4141 Munchenstein, Switzerland; nbolog@phoenixswissmed.com; 4 Department of Medical Informatics and Biostatistics, “Iuliu Haţieganu” University of Medicine and Pharmacy, 400349 Cluj-Napoca, Romania; dleucuta@umfcluj.ro; 5 Department of Cranio-Maxillofacial Surgery, “Iuliu Haţieganu” University of Medicine and Pharmacy, 400001 Cluj-Napoca, Romania; hrotaru@elearn.umfcluj.ro; 6 Department of Dental Medicine, Faculty of Medicine and Pharmacy, University of Oradea, 410563 Oradea, Romania; 7 Department of Prosthodontics, “Iuliu Haţieganu” University of Medicine and Pharmacy, 400006 Cluj-Napoca, Romania; dana.buduru@umfcluj.ro

**Keywords:** temporomandibular joint, effusion, magnetic resonance imaging, ultrasonography

## Abstract

Aim: The aim of this study was to evaluate if the increased temporomandibular joint (TMJ) capsular thickness, measured by ultrasound (US), is associated with the presence of effusion, diagnosed using MRI imaging. Materials and Methods: 102 patients with signs and symptoms of temporomandibular disorders were included in the study. Each patient underwent US and MRI examinations, 1 to 5 days following clinical examination. The US was performed with an 8–40 MHz linear transducer operating at 20 MHz. The MRI was performed using a 1.5 T MRI device. The ROC curve was analyzed to identify the optimal cut-off value for capsular distention, which can be interpreted as an indirect sign of TMJ effusion. Results: The capsular width values were found to be between 0.7 and 3.6 mm. The best cut-off value was 2.05 mm with a sensitivity of 55.9% and a specificity of 94.7%. The next optimal cut-off value was 1.75 mm with a sensitivity of 67.6% and a specificity of 82.4%. The area under the ROC curve was 0.78 (95% CI 0.68, 0.87, *p* < 0.05). Conclusions: Ultrasound-measured capsular width can be interpreted as an indirect sign of TMJ effusion. The critical cut-off for capsular width was 2 mm.

## 1. Introduction

Fluid accumulation (effusion) inside the temporomandibular joint (TMJ) is a consequence of inflammation of the synovial membrane, with excessive production of synovial fluid [1]. This can occur as a result of major trauma of the facial structures, repeated micro-traumas, or different occlusal conditions [2]. Repeated micro-trauma can be generated by prolonged muscle hyperactivity (bruxism, clenching) or by mandibular orthopedic instability [2,3]. There are also general etiological factors responsible for the inflammation of the TMJ, the most common being rheumatoid arthritis, psoriatic arthritis, and ankylosing spondylitis [4].

The presence of synovitis and intra-articular fluid influences the therapeutic scheme; therefore, identifying inflammatory changes is very important. From a clinical point of view, this situation is frequently associated with joint pain, although the inflammatory changes in the TMJ are not always correlated with symptom severity [3].

Currently, the gold standard for the diagnosis of joint effusion is magnetic resonance imaging (MRI), which is the only examination able to reveal early phases of inflammation represented by subchondral edema [4,5,6]. The use of MRI is sometimes limited due to high costs and lack of availability. Therefore, the need for alternative techniques has increased. Ultrasonography (US) is reported in the literature as a simple, non-invasive, dynamic, and inexpensive technique for assessing TMJ pathology in terms of disc position, degenerative changes, and effusion [7,8,9,10,11,12,13,14,15,16].

The aim of this study was to evaluate if the increased capsular thickness, highlighted and measured by US, is associated with the presence of joint effusion, diagnosed using MRI imaging.

Another objective was to establish the critical value of high-resolution ultrasound-measured capsular distension as an indirect marker for joint effusion.

## 2. Materials and Methods

### 2.1. Patients

A group of 102 patients (204 TMJs) was included in this prospective study. The patients were referred to the clinic for TMJ disorders. The inclusion criteria were signs and symptoms of TMJ internal derangements, according to research diagnostic criteria for temporomandibular disorders (RDC/TMD) [17]. Exclusion criteria were contraindications to MRI examination (claustrophobia and ferromagnetic metal carriers), patients with pure muscular disorders, patients with systemic disease, and patients undergoing anti-inflammatory drug treatment [9]. Patients in which misaligned images or distortions due to motion artefacts made the interpretation of the MRI unreliable were excluded. 

Clinical examination was performed by an oral surgery specialist and consisted of analyzing the TMJ, the masticatory and cervical muscles, and the dental occlusion. The examination of all masticatory muscle groups, both endo-oral and exo-oral, was performed in order to make a differential diagnosis between a muscular and an articular disease. 

Each patient underwent US and MRI examination, 1 to 5 days following clinical examination.

All methods were carried out in accordance with the relevant guidelines and regulations. Informed consent was obtained from each participant included in this study. For subjects under the age of 18, informed consent was obtained from a parent and/or legal guardian. 

Ethical approval (no. 403/02.07.2015) for the study was obtained from the Ethical Committee of the University of Medicine and Pharmacy “Iuliu Hatieganu” Cluj-Napoca. 

### 2.2. MRI Examination

All the patients included in the study were evaluated using a 1.5 Tesla MRI device in the same multichannel head coil. T1-weighted, T2-weighted fat suppression, and proton density (PD) sequences obtained in the oblique sagittal and coronal planes, in closed- and opened-mouth positions, perpendicular and parallel to the long axis of the condyle were included in the acquisition protocol [18]. 

**Figure 1:**
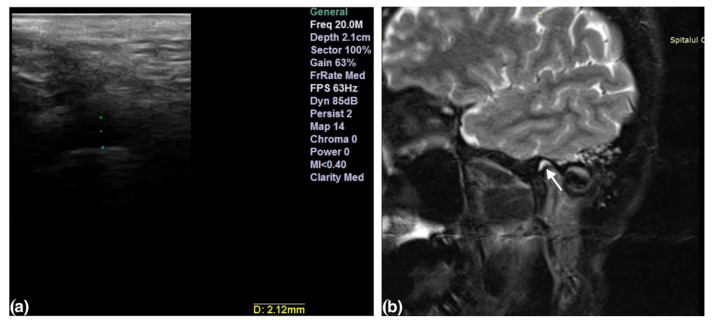
High-resolution 20 MHz US image (**a**) of joint effusion detected by direct visualization and abnormal capsular width (2.12 mm). Sagittal oblique T2-weighted TSE with fat suppression image (**b**) of the same joint with effusion (arrow) posterior to the disc in the closed-mouth position.

**Figure 2:**
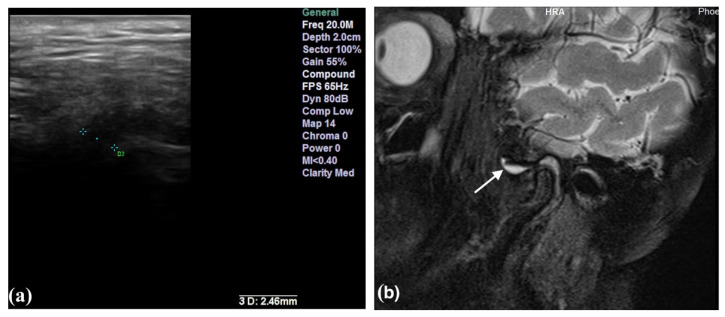
High-resolution 20 MHz US image (**a**) of joint effusion detected by direct visualization and abnormal capsular width (2.46 mm). Sagittal oblique T2-weighted TSE with fat suppression image (**b**) of the same joint with effusion (arrow) anterior to the disc in the closed-mouth position.

The presence of effusion and disc displacement were assessed in all the TMJs. Joint effusion was established by identifying thin lines or a collection of high signal intensities inside the articular space in T2-weighted sequences (Figure 1b and Figure 2b). Disc displacement was confirmed when the posterior margin of the posterior band was situated anteriorly to the vertical orientation of the condyle (the twelve o’clock line). The sideways-dislocated disc was seen most clearly in the oblique coronal sequences and was present when the disc crossed over one of the lines through the condylar poles [19]. 

Synovitis was also detected as a thickening of the synovial membrane (diffused or irregular) [5]. Contrast-enhanced MRI examinations may be useful as an additional tool in some cases (e.g., accurate measurement of the synovial membrane). However, for the detection of intra-articular effusion, as well as for the distribution of the fluid around the disc, T2-weighted images deliver sufficient information for routine clinical imaging. Therefore, the standard MRI protocol does not include the intravenous contrast administration, and the contrast is not routinely administered to patients with TMJ disorders. In some chronic cases, synovial fluid may appear inhomogeneous due to internal foreign bodies of a few millimeters (“rice bodies”) [4].

### 2.3. The Ultrasound Examination

**Figure 3:**
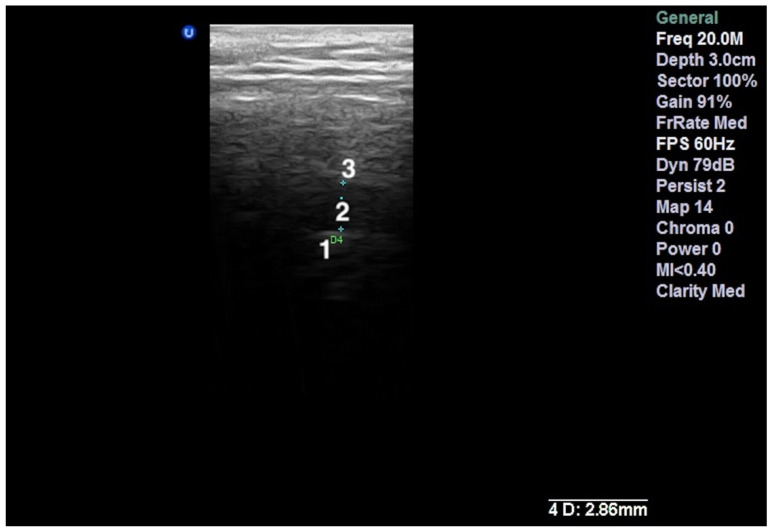
High-resolution 20 MHz US image of an effusion in the left TMJ detected by abnormal capsular width (2.86 mm). 1—mandibular condyle; 2—articular disc; 3—glenoid fossa.

The ultrasound examinations were performed on a Sonotouch and tablet system machine, with a linear transducer with a variable frequency between 8 and 40 MHz. An examiner with 10 years of experience in maxillofacial ultrasonography evaluated all the patients, using the 20 MHz frequency. The transducer was placed perpendicular to the zygomatic arch and parallel with the vertical ramus of the mandible, corresponding to the anatomical location of the TMJ. The images were obtained in the transversal and longitudinal planes. The intra-articular fluid was depicted either directly by visualization of a marked hypoechoic area in the joint or by measuring the capsular width between the superior condylar surface and the most lateral point of the articular capsule [8]. All the measurements were performed in the closed-mouth position (Figure 1a and Figure 2a). The capsular width was measured in millimeters (mm). The articular bone surfaces, represented by the articular eminence and the mandibular condyle, were identified as two hyperechoic arcuate lines (Figure 3). 

**Figure 4:**
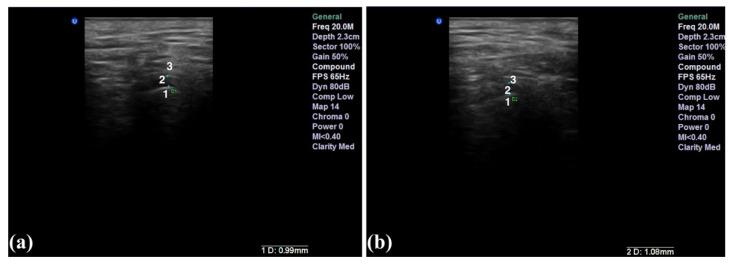
High-resolution 20 MHz US image of a normal TMJ: closed-mouth (**a**), opened-mouth (**b**). 1—mandibular condyle; 2—articular disc, situated with the intermediate part between the anterosuperior zone of the mandibular condyle and the posterosuperior part of the articular eminence; 3—glenoid fossa.

At US examination, the disc is described as a hyperechoic line surrounded by a hypoechoic area [20]. Normally, the disc is located between the two anatomical landmarks mentioned below (Figure 4). The anterior or posterior position, when compared to the normal, anatomical location, is defined as disc displacement.

Both examiners were blinded regarding the clinical examination and the other imaging results.

### 2.4. Statistical Analysis

The receiver operator characteristic curve (ROC) was plotted for the presence of the joint effusion identified with MRI, using the capsular width (mm) as measured by 20 MHz US. Its chart was plotted along with a 95% confidence interval computed by bootstrapping. The best cut-off was computed by identifying the best Youden index (sensitivity + specificity—1). A table with all the cut-off values along with all sensitivities and specificities was computed. All statistical analysis was carried out with the R environment for statistical computing and graphics (R Foundation for Statistical Computing, Vienna, Austria), version 3.4.3 [21]. 

## 3. Results

The study sample consisted of 102 subjects (204 TMJs), with a median age of 29 years (range 13–69 years). Of the total subjects included in the study, 84 were females.

**Figure 5:**
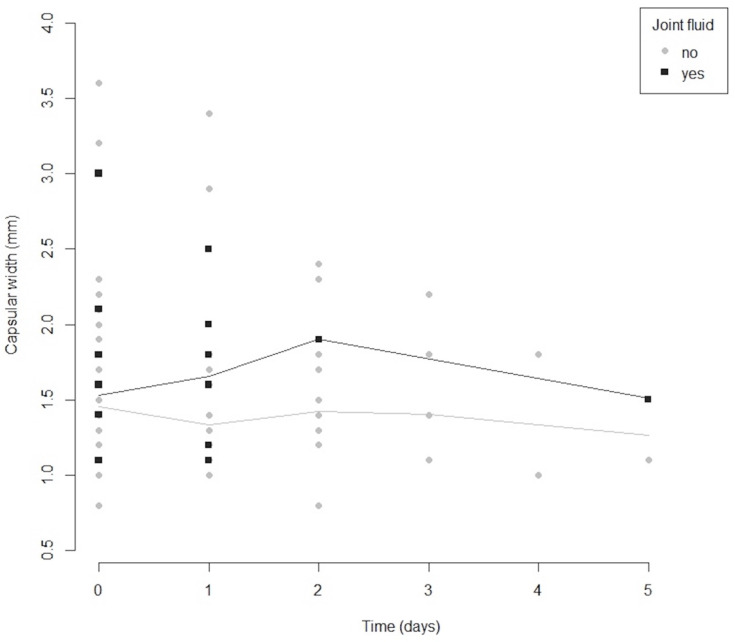
Scatterplot chart showing the relation between inter-observation time (MRI and US) and the capsular width, along with locally estimated scatterplot smoothing lines.

The time median (IQR) between the two examinations (MRI and US) was 1 (0–2), ranging between 0 and 5 days. To assess if there was a relation between the inter-observation time (the time between the two imaging examinations) and the capsular width, we used a scatterplot chart (Figure 5). The distribution of points and the locally estimated scatterplot smoothing lines did not indicate any association. Furthermore, there was no statistically significant correlation between the two variables (Spearman correlation coefficient = −0.06, *p* = 0.56).

**Figure 6:**
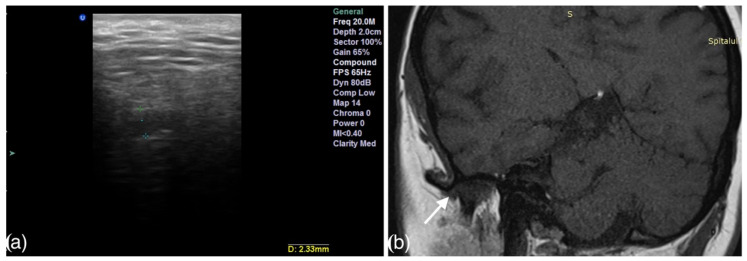
High-resolution 20 MHz US image of a TMJ with abnormal capsular width (2.33 mm) (**a**). Coronal T1 closed-mouth image (**b**) of the same TMJ with lateral disc displacement (arrow).

Out of 102 patients enrolled in this study, 41 presented bilateral signs and symptoms of TMJ internal derangements. The capsular thickness values ranged between 0.7 and 3.6 mm and are shown in Table 1, together with the presence/absence of joint effusion diagnosed using MRI. According to MRI, 34 joints presented effusion. MRI showed effusion in 11% of the joints with normal disc position. The remaining joints (89%) with effusion presented disc displacement. From the total number of examined joints, 4 presented pure lateral disc displacement (Figure 6).

**Figure 7:**
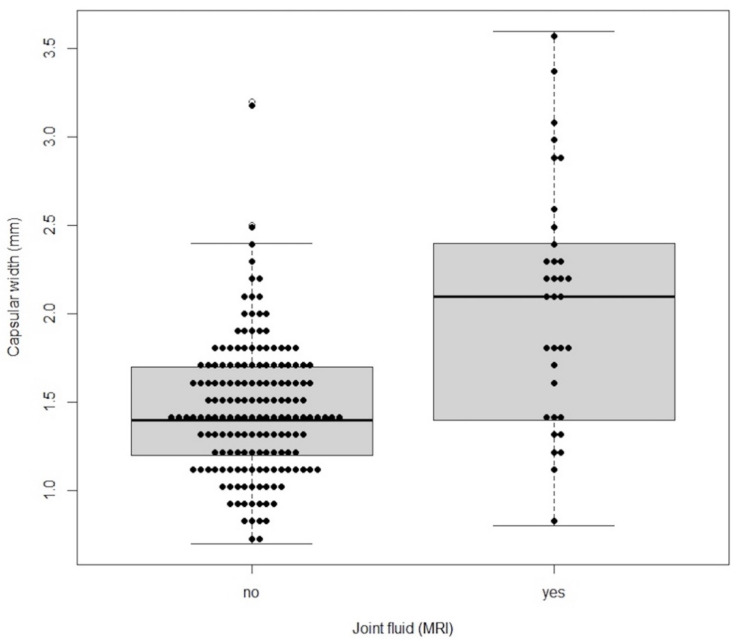
Bee swarm plot along with boxplot of the distribution of capsular width with values grouped by joint effusion presence.

The capsular width median (IQR) for normal values (without joint effusion) was 1.4 mm (1.2–1.7), ranging between 0.7 mm and 3.2 mm. The capsular width median (IQR) for abnormal values (with joint effusion) was 2.1 mm (1.45–2.375), ranging between 0.8 mm and 3.6 mm. The distribution of capsular width values as an indication of the presence or absence of joint effusion is shown in Figure 7. 

**Figure 8:**
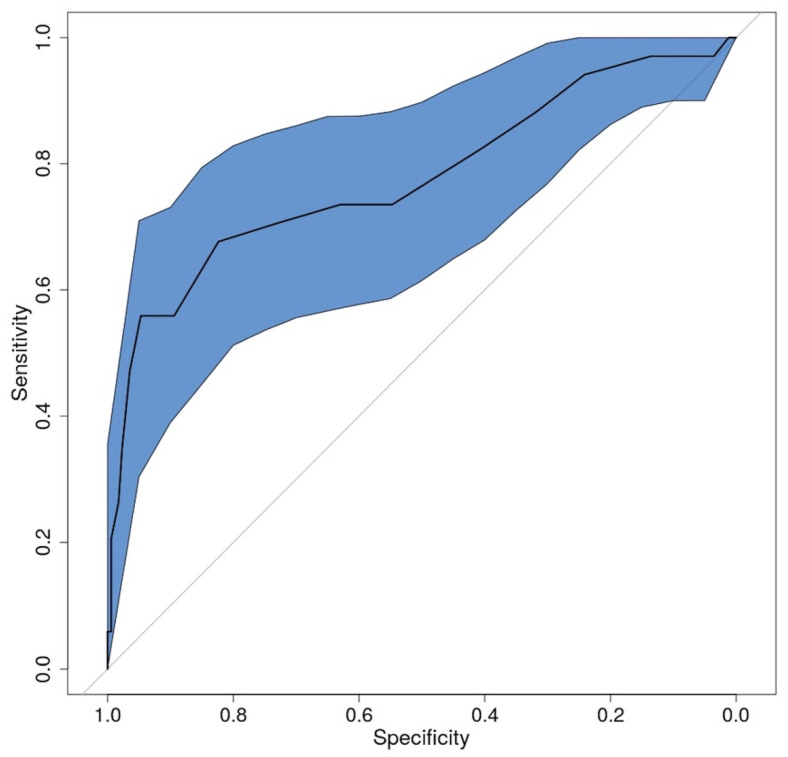
The receiver operator characteristic curve (ROC) was plotted for the presence of the joint fluid identified with MRI, using the capsular width (mm) as measured by high-resolution 20 MHz US.

A ROC curve was created in order to establish the most accurate value of capsular width to distinguish between TMJs with and without effusion (Figure 8). The area under the ROC curve (AUC) was 0.78 (95% CI 0.68, 0.87, *p* < 0.05). 

A list displaying all cut-off values, as well as their corresponding sensitivities and specificities, is presented in Table 2. The critical area is around 2 mm. The best cut-off value for capsular distension is 2.05 mm, with a sensitivity of 55.9% and a specificity of 94.7%. The next best cut-off value is 1.75 mm, with a sensitivity of 67.6% and a specificity of 82.4%.

## 4. Discussion

US is successfully used to identify inflammatory changes in large joints [22,23]. It is a noninvasive, less expensive technique than MRI. The use of US for TMJ exploration is currently controversial in the literature [8,9]. This is due to the fact that US is a method that strictly depends on the operator and on the frequency of the transducer that is being utilized. High-frequency transducers have improved the diagnostic quality of US [24,25]. US is an easy technique to diagnose TMJ disc displacements. In identifying the position of the disc, there are authors who argue that US is a useful diagnostic method, while others have shown that the diagnostic accuracy of US is relatively low [8,9,11,24]. 

The detection of inflammatory changes in the TMJ (synovitis and effusion) can be carried out directly with US, by identifying the fluid collection as a hypoechoic area, or indirectly, by quantifying the degree of capsular distension. Very small differences (0.2–0.3 mm) in measuring capsular distension significantly influence US sensitivity and specificity as a diagnostic test for TMJ effusion [7].

MRI investigation is the standard reference in the diagnosis of inflammatory diseases of the TMJ [4]. Changes in the TMJ that occur in inflammatory diseases have similar manifestations as in other joints: subchondral bone modifications and synovial collection with synovitis [26]. Bone pathological changes are represented by subchondral edema, subchondral erosions, and bone resorption with shape modifications of the condyle and of the temporal fossa [27,28]. MRI examination is the gold standard for diagnosing subchondral edema, which is one of the most important signs in the early stages of the disease [29]. Although not pathognomonic (it can also occur in mechanical dysfunctions), subchondral edema is the result of inflammatory osteitis that occurs in the acute phase and precedes the onset of subchondral erosions [27]. The MRI examination shows a diffused T2 hypersignal in the subchondral bone marrow. Early signs of TMJ inflammation, such as subchondral oedema or minimal synovial thickening, can be found in T2-weighted sequences [30].

The AUC obtained in this study indicated good diagnostic accuracy when using US to identify capsular width as an indirect marker of fluid collection in the TMJ. The optimal cut-off value of the capsular distension was 2.05 mm, with a sensitivity of 55.9% and a specificity of 94.7%. The second optimal threshold value was 1.75 mm, with a sensitivity of 82.4% and a specificity of 67.6%. Sensitivity increased at values below the optimum threshold of 2.05 mm, while specificity increased at values above 2.05 mm.

The optimal threshold value obtained in our study was consistent with that obtained by Manfredini et al. [31]. Bas et al. [32] identified a threshold value of capsular distension of 1.65 mm. The differences between these values are due to the operator-dependent character of US and the lack of standardization of US examination of TMJ. 

The diagnostic accuracy of US in the detection of TMJ effusion has been evaluated by several authors. Manfredini et al. [31] obtained an Se of 80%, while Jank et al. [14] obtained an Se of 81%. Tognini et al. [33] reported a good diagnostic accuracy of US, studying the ROC curve. Melchiore et al. [7] reported an Se of 70.6%. All these studies used MRI investigation as a gold standard.

The capsular width median obtained in the present investigation was 1.4 mm, with a range between 0.7 mm and 3.2 mm. The high values were seen in the cases of lateral disc displacements. Elias et al. [34] attempted to establish normal values of lateral capsular thickness. This value ranged between 1.4 mm and 1.6 mm. 

Capsular thickness may increase in the case of lateral displacements of the articular disc (Figure 6). Strictly lateral discal displacements are less common [3]. In the present study, MRI detected four joints with lateral disc displacement. In these situations, it is difficult to make a differential ultrasonographic diagnosis between effusion and lateral disc displacement and, usually, the measured joint space distension is thicker. However, joint pain may be a sign of a differential diagnosis in such cases. Synovitis and fluid accumulation inside the TMJ are often accompanied by pain. A correlation between joint pain, capsular distension, and the presence of joint fluid confirmed by MRI was made in the study of Bas et al. [32]. A positive correlation was found between pain scores and the degree of fluid accumulation confirmed by MRI. The optimal threshold value of the ultrasound-measured capsular distension obtained by this group of researchers was 1.65 mm. The link between joint pain and the presence of fluid collection in the TMJ, diagnosed with MRI imaging, has been investigated by several authors, most obtaining a close link between pain and the presence of intra-articular fluid [5,35,36,37]. Other authors [38,39] concluded that there is no link between joint pain and intra-articular fluid accumulation.

The strength of this study lies in the large number of joints examined (204) and the short time in which the clinical examinations, US and MRI, were performed. The time between the two measurements (MRI and US) was short, ranging from 0 to 5 days, 73% within one day and 18% within two days, thus unlikely introducing an observation bias. Moreover, we could not observe any relation between inter-observation time and capsular width, thus another argument that the time between measurements did not induce an information bias (Figure 5). In other studies [31,32,33], the interval between MRI and US was 2–3 weeks. A large chronological difference between the two investigations may influence the results of the study. Inflammatory changes may remit or, on the contrary, may intensify. 

The limitation of US imaging is mainly represented by the difficulty of visualizing the TMJ components in the opened-mouth position due to the overlapping of bony structures. This depends on the examiner’s skills and experience. 

Another limitation of the study is that no sample size calculation was performed. We used a consecutive sample of all patients with the selection criteria, thus, the best non-probabilistic sampling possible in our setting. Therefore, we consider our cohort to be representative for this type of study.

Scarce research on this topic suggests the need for conducting new studies to assess intra- and inter-observational variability. It was found that for minor differences in threshold values for capsular width, sensitivity, and specificity can substantially change. For example, a cut-off value of 1.85 mm showed a sensitivity of 55.9% and a specificity of 89.4%, while a cut-off value of 1.65 mm showed a sensitivity of 70.6% and a specificity of 72.9%. This could affect the diagnostic accuracy of US in cases of TMJ effusion. These minor differences of 0.2 to 0.3 mm may be due to inter- and intra-observational variability. The evaluation of this variability is difficult to achieve using only retrospective analysis of static images. For this reason, storage and analysis of videos, which can provide more information for the examiner, is recommended.

High-resolution US did not reach the diagnostic value of MRI in the assessment of TMJ inflammatory changes, but it remains a dynamic, simple, and less expensive technique with good diagnostic accuracy that can be used for screening patients with suspected effusion. Nevertheless, MRI remains the preferred examination for this pathology. However, in some regions, there is a lack of availability of MRI equipment, or it is an expensive technique. In these situations, US can provide valuable information regarding the TMJ status. 

## 5. Conclusions

According to the present study, capsular distension can be interpreted as an indirect sign of TMJ effusion. The critical area for capsular width is around 2 mm. 

Differential ultrasonographic diagnosis between lateral disc displacement and joint effusion is difficult to achieve. In both cases, there is significant capsular distension. 

## Figures and Tables

**Table 1:** Capsular width values and the presence/absence of joint effusion confirmed by MRI.

	Joint Effusion—MRI		
	Yes	No	Total
**Capsular Thickness (mm)-US**			
0.7	0	2	2
0.8	1	4	5
0.9	0	7	7
1	0	10	10
1.1	1	18	19
1.2	2	13	15
1.3	2	15	17
1.4	3	24	27
1.5	0	14	14
1.6	1	17	18
1.7	1	16	17
1.8	4	12	16
1.9	0	5	5
2	0	4	4
2.1	3	3	6
2.2	4	2	6
2.3	3	1	4
2.4	1	1	2
2.5	1	1	2
2.6	1	0	1
2.9	2	0	2
3	1	0	1
3.1	1	0	1
3.2	0	1	1
3.4	1	0	1
3.6	1	0	1
Total	34	170	204

MRI—magnetic resonance imaging, US—ultrasonography.

**Table 2:** Capsular width cut-off value (mm) along with the corresponding sensitivities and specificities.

	Capsular Width Cut-Off Value (mm)	Sensitivity	Specificity
1	−Inf	100.00	0.00
2	0.75	100.00	1.20
3	0.85	97.10	3.50
4	0.95	97.10	7.60
5	1.05	97.10	13.50
6	1.15	94.10	24.10
7	1.25	88.20	31.80
8	1.35	82.40	40.60
9	1.45	73.50	54.70
10	1.55	73.50	62.90
11	1.65	70.60	72.90
12	1.75	67.60	82.40
13	1.85	55.90	89.40
14	1.95	55.90	92.40
15	2.05	55.90	94.70
16	2.15	47.10	96.50
17	2.25	35.30	97.60
18	2.35	26.50	98.20
19	2.45	23.50	98.80
20	2.55	20.60	99.40
21	2.75	17.60	99.40
22	2.95	11.80	99.40
23	3.05	8.80	99.40
24	3.15	5.90	99.40
25	3.30	5.90	100.00
26	3.50	2.90	100.00
27	Inf	0.00	100.00
